# Ultra-Sensitive HIV-1 Latency Viral Outgrowth Assays Using Humanized Mice

**DOI:** 10.3389/fimmu.2018.00344

**Published:** 2018-03-05

**Authors:** Kimberly Schmitt, Ramesh Akkina

**Affiliations:** ^1^Department of Microbiology, Immunology and Pathology, Colorado State University, Fort Collins, CO, United States

**Keywords:** HIV-1 latent viral outgrowth assay using humanized mice, humanized mouse-based HIV-1 latency outgrowth assay, comparison of quantitative viral outgrowth assays with humanized mouse-based viral outgrowth assay, comparison of mVOA with humanized mouse-based viral outgrowth assay, non-human primate-based latent simian immunodeficiency viral outgrowth assay, sensitivity of humanized mouse-based viral outgrowth assay over mVOA, ultra-sensitive HIV-1 latent viral outgrowth assay in hu-mice, mouse-based HIV-1 viral outgrowth assays

## Abstract

In the current quest for a complete cure for HIV/AIDS, highly sensitive HIV-1 latency detection methods are critical to verify full viral eradication. Until now, the *in vitro* quantitative viral outgrowth assays (qVOA) have been the gold standard for assessing latent HIV-1 viral burden. However, these assays have been inadequate in detecting the presence of ultralow levels of latent virus in a number of patients who were initially thought to have been cured, but eventually showed viral rebound. In this context, new approaches utilizing *in vivo* mouse-based VOAs are promising. In the murine VOA (mVOA), large numbers of CD4^+^ T cells or PBMC from aviremic subjects are xenografted into immunodeficient NSG mice, whereas in the humanized mouse-based VOA (hmVOA) patient CD4^+^ T cell samples are injected into BLT or hu-hematopoetic stem cells (hu-HSC) humanized mice. While latent virus could be recovered in both of these systems, the hmVOA provides higher sensitivity than the mVOA using a fewer number of input cells. In contrast to the mVOA, the hmVOA provides a broader spectrum of highly susceptible HIV-1 target cells and enables newly engrafted cells to home into preformed human lymphoid organs where they can infect cells *in situ* after viral activation. Hu-mice also allow for both xenograft- and allograft-driven cell expansions with less severe GvH providing a longer time frame for potential viral outgrowth from cells with a delayed latent viral activation. Based on these advantages, the hmVOA has great potential in playing an important role in HIV-1 latency and cure research.

## Introduction

Since the beginning of the deadly HIV/AIDS epidemic, major research emphasis has been placed on developing effective vaccines for prevention and potent drugs to control the infection. Since HIV-1 is a retrovirus which integrates into the host cell genome and can establish viral latency, a complete cure was thought not to be possible until the case of the “Berlin patient” (1, 2). This HIV-1+ patient had undergone allogenic bone marrow (BM) transplantation from a homozygous *CCR5*Δ*32* donor to treat acute myeloid leukemia. No HIV-1 could be detected during later years in this individual even after extensive testing thus confirming his HIV-1 negative status and a complete cure. Following this example additional cases of possible HIV-1 cure generated excitement.

Two individuals known as the “Boston patients,” (A and B) underwent allogeneic hematopoietic stem cell transplant (HSCT), in this case with wild-type CCR5+ donor cells to treat lymphoma (3). For 4.3 years after the transplant both patients were treated with ART (4). During this time no proviral DNA or replication-competent virus could be detected in PBMC, plasma or rectal tissues by using the most sensitive methods including the gold standard quantitative viral outgrowth assays (qVOA)(3, 4). After the cessation of ART however, virus rebounded within patient A by 12 weeks and patient B by 32 weeks (5). In the case of the “Mississippi baby,” ART was started 30 h after birth and continued for the first 18 months of life (6). After the cessation of ART, the “Mississippi baby” controlled viremia for 2 years and was antibody negative (7). No HIV-1 could be detected with PCR tests or qVOA using 22 million resting CD4^+^ T cells (6, 7), which led to the speculation that she could be another example of a complete HIV-1 cure. However, the virus eventually rebounded. Both of these cases exemplified “potential cures,” wherein all the tests including the gold standard qVOA (see below) could not detect the ultralow levels of latently infected cells thus necessitating the search for more sensitive HIV-1 latency detection methods.

### Current Assays for Measuring the Latent Viral Reservoir and Limitations

Since the latent HIV-1 is transcriptionally silent and the minuscule number of latently infected cells (0.1–10 infectious units per million (IUPM) resting CD4^+^ T cells) are distributed over difficult to reach anatomical sites measuring the quiescent viral reservoir poses challenges (8–12). Many sensitive viral DNA, RNA or protein detection methods are currently employed to determine the viral burden (13). However, they overestimate the reservoir size as they cannot distinguish between the defective viral genomes and replication-competent virus. More recent advanced assays could simultaneously assess viral RNA, proteins and cell markers enabling the detection of viral induced cells (14–18). However, limitations remain to distinguish and accurately measure the true replication-competent latent virus.

The most accurate approach in determining the full efficacy of HIV-1 cure strategies is analytic treatment interruption (ATI) also known as monitored antiretroviral pause. However, this is impractical for routine application and poses unnecessary risk. The long-standing qVOA is considered as the “gold standard” in the HIV-1 latency field to measure the replication-competent virus and employs a co-culturing method to amplify the induced virus from rare latent cells (19–21). Serial dilution of test cells allows for quantitation, expressed as IUPM (22). Besides being time-consuming, a major drawback with this method is its tendency to underestimate the viral reservoir size since not all latent cells are induced during the assay period (23). New versions of the qVOA have been developed that use reporter cells and/or cell lines to amplify the virus with significantly increased sensitivity in a shorter time-span (13–17, 24, 25). Importantly, the stochastic aspect observed during *in vitro* viral activation wherein repeated stimulation of cells over time results in release of virus from previously non-responding cells (23) suggesting that approaches such as the *in vivo* methods described below that allow for long-term viral outgrowth may capture these late responder cells.

### Non-Human Primate (NHP) Models of Latency Detection

Many aspects of HIV-1 pathogenesis and latent reservoirs distributed in different anatomical sites are difficult to directly assess if not impossible to study in a human subject. In this context, the simian immunodeficiency (SIV)-macaque model of AIDS has been extremely useful in gathering relevant data on viral persistence and latency (26–28). In NHP studies, latent virus was successfully recovered from naïve macaques that underwent adoptive transfer of resting CD4^+^ T cells obtained from virally suppressed SIV-infected macaques (as determined by all standard tests) undergoing intensive ART (29). These findings showed that ultralow levels of otherwise undetectable latently infected cells could be induced and detected with an *in vivo* system using adoptive transfer of test cells. More recently, Avalos et al. assessed viral persistence in brain macrophages of five ART-suppressed SIV-infected pig-tailed macaques using a newly developed macrophage quantitative viral outgrowth assay (Mϕ-VOA) (30). In one macaque, latency reversing agents (LRAs) ingenol-B (protein kinase C agonist) and vorinostat (HDAC inhibitor) reactivated latent viral genomes that were genetically distinct from virus circulating in the plasma. This data demonstrated the utility of the Mϕ-VOA for latency detection in macrophages.

### Non-Humanized Mouse Models for Latent Viral Outgrowth (mVOA)

**Figure 1:**
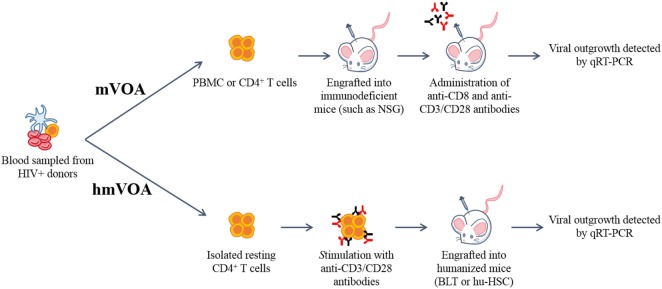
Schematic representation of the murine (mVOA) and humanized mouse-based viral outgrowth assay (hmVOA) for HIV-1. Previously frozen unfractionated PBMC or isolated resting CD4^+^ T cells obtained from HIV-1 infected donors on ART with undetectable viral loads were used for both the mVOA and hmVOA. In the mVOA, cells were either clonally expanded or directly xenografted into immunodeficient mice (such as NSG). CD8^+^ T cells were depleted and T cell activation was prolonged using anti-CD3/CD28 antibodies *in vivo*. In the hmVOA, resting CD4^+^ T cells were purified using PBMC and stimulated with anti-CD3/CD28 antibodies. Stimulated cells were xenografted into either hu-HSC or BLT mice. Viral outgrowth was detected by qRT-PCR.

Immunodeficient mice permit the transplantation of human cells such as PBMCs without rejection which led to the development of the hu-PBL-SCID mouse model (31, 32). Infection of these mice with HIV-1 gives rise to viremia and the engraftment of PBMC from HIV-1+ subjects resulted in viral outgrowth. With this as a background, Metcaf Pate et al. recently developed a latent HIV-1 murine viral out growth assay (mVOA) (Figure 1) (33). The mVOA assay is based on the principle that engrafted human cells undergo xenograft-mediated expansion leading to consequent latent viral induction. Immunodeficient NSG mice were injected with large number of cells (66 million PBMC or 10–26 million resting CD4^+^ T cells) from 11 HIV-1+ subjects, including six elite controllers. All of these samples had undetectable viral loads by qRT-PCR (<50 copies/mL) but were positive for viral outgrowth by qVOA except for one elite controller. The engrafted mice were treated with anti-CD8 antibody to deplete the human CD8^+^ T cells and with anti-CD3/CD28 antibodies for the activation of T cells. Viral outgrowth was detected in all 11 patient samples in these mice, including the one elite controller negative for viral outgrowth in the qVOA. This study also evaluated latent cells from ART-suppressed SIV-infected pig-tailed macaques. NSG mice were injected with 40 million PBMC or 6.8 million resting CD4^+^ T cells. All inoculated mice had detectable SIV RNA in the plasma after 7 days.

Another recent mVOA study evaluated cells from two HIV-1 infected subjects enrolled in a PrEP program who were treated soon after infection (participants A and B treated within 10 and 12 days, respectively) (34). During the following 2-year period, participant A had undetectable HIV RNA and/or DNA in both the blood and tissue whereas participant B showed a low level of intermittent HIV RNA and/or DNA in various CD4^+^ T cell subsets, but not in tissue samples. The qVOA results were negative. To test these patients’ cells for latent viral detection by mVOA, 530 million peripheral CD4^+^ T cells from participant A (53 million per mouse, 10 mice total) and 379 million cells from participant B (50 million per mouse, eight mice total), were injected intraperitoneally into NSG mice. Approximately 5.5 weeks post-inoculation, mice were treated with anti-CD3 antibody to stimulate T cells *in vivo* and reactivate latent virus. One out of ten mice injected with CD4^+^ T cells from participant A became borderline positive (201 copies per ml) at only one time point. Terminal mouse spleen tissue sample was negative for viral detection and both RNA and DNA sequencing efforts for viral identification by an independent laboratory were unsuccessful. In contrast, three out of eight mice injected with CD4^+^ T cells from participant B became strongly virus positive with high viral loads (1,000, 5,000 and 11,000 copies per ml). While the sample sizes of the qVOA negative subjects are small in the above two studies, it is apparent that the mVOA could recover latent virus to a certain extent (2 out of 3 samples).

In a different twist to the mVOA, Yuan et al. utilized cells from a single aviremic subject which were positive for viral outgrowth by *in vitro* qVOA (0.518 IUPM) (35). First, the subject’s CD4^+^ T cells were clonally expanded *in vitro* and then split into two groups: qVOA negative or positive. NSG mice were then injected with resting or clonally expanded CD4^+^ T cells from each group. The clonally expanded cells that appeared qVOA positive and used to inoculate mice displayed detectable HIV-1 within 4 weeks while the qVOA negative cells used to inject mice became positive by week 10. Utilization of split portions of clonally expanded cells with a potentially uneven distribution of qVOA positive cells in the test samples, sample size of a single patient and lack of details on how many mice were used are limitations of this study.

In a recent report by Salgado et al., CD4^+^ T cells isolated from four HIV-1+ subjects that underwent allogenic BM stem cell transplantation to treat hematalogic malignancies were evaluated for the presence of any residual latent virus (36). Five immunodeficient NSG mice per each donor were xenografted with 10–50 million cells to detect possible viral outgrowth. However, none of these xenografted mice showed positive viral outgrowth by week 13. Since it is unlikely that these four individuals are fully cured based on previous examples like the “Boston patients,” and mVOA was not able to recover any latent virus from these, caution needs to be exercised about the reliability of mVOA for ultra-sensitive latency detection.

Several other limitations also exist for mVOA in its current form (Table 1). These include variable levels of donor cell engraftment, the need for CD8^+^ T cell depletion through injection of anti-CD8 antibodies and the administration of anti-CD3/CD28 antibodies for prolonged T cell activation. Most importantly, since a very large number of donor cells are xenografted, rapid GvH is a major drawback often resulting in untimely/unpredictable loss of engrafted mice thus not permitting longer assay periods to allow for the detection of delayed latent virus outgrowth.

**Table 1:** The advantages and disadvantages of the mVOA and humanized mouse VOA (hmVOA) for HIV-1 latency detection.

Model	Methods	Advantages	Disadvantages
mVOA	Large number of human PBMC or CD4^+^ T cells are xenografted into NSG mice followed by the administration of anti-CD8 and anti-CD3/CD28 antibodies	Straightforward inoculation of donor cells into NSG mice Larger number of cells can be assayed compared to the *in vitro* quantitative viral outgrowth assays (qVOA) Can be used to assess either HIV-1+ or SIV+ donor samples	Rapid onset of GvH, thus limiting the assay’s time table Variable levels of donor cell engraftment Additional anti-CD8 and anti-CD3/CD28 antibody injections are needed

hmVOA	Resting CD4^+^ T cells are xenografted into humanized mice	Broader spectrum of HIV-1 target cells are available Engrafted cells home into a preexisting lymphoid system Allows for both xeno- and allograft-mediated stimulation and cell expansion Less severe (BLT mice) or no GvH (hu-HSC mice) No additional antibody (anti-CD8 and anti-CD3/CD28 antibody) injections required Larger number of cells than the qVOA can be assayed and fewer number of cells required than the mVOA	Human hematopoetic stem cells (HSC) and tissues are required to prepare the humanized mice More expensive

### Humanized Mouse Model-Based Latent Viral Outgrowth Assay (hmVOA)

New generation humanized mouse models have now become integral tools in many aspects of HIV research. The advent of highly immunodeficient mice incorporating the IL-2 receptor common gamma chain (IL2Rγc) mutation together with others, such as SCID, NOD, RAG1, or RAG2 gene mutations permitted far superior human tissue/cell engraftment (31, 37). Among these are the Rag1^−/−^ γc^−/−^, Rag2^−/−^γc^−/−^, NOD/Shi-scid/γc^−/−^ null (NOG), NOD/SCIDγc^−/−^ (NSG), NOD.Rag1KO.IL2RγcKO (DRAG), and NOD.HLA-A2.HLA-DR4.RagKO.IL2RγcKO (DRAGA) (38, 39). Two current leading hu-mouse models are the hu-HSC and BLT mice. Hu-HSC mice are prepared by intrahepatic injection of CD34^+^ HSC into irradiated newborn RAG1, RAG2, NSG or NOG mice (40–43). Engraftment of these mice seeds the BM and gives rise to *de novo* multilineage human hematopoiesis. BLT mice are prepared by surgical implantation of human fetal liver and thymic tissue under the kidney capsule in addition to reconstitution with autologous HSC (40, 42, 44, 45). In both these models, there is *de novo* production of human T cells, B cells, monocytes/macrophages, dendritic cells and NK cells, as well as successful mucosal compartment engraftment (40–42, 45). While both the models permit human immune responses, the presence of an autologous human thymus in BLT mice allows for human T cell education and HLA restricted responses (40, 42–47). Thus, these hu-mice offer an excellent *in vivo* system for the engraftment and long-term maintenance of exogenous latently infected cells and potential outgrowth of the latent virus from these. Another potentially suitable hu-mouse model currently available employs HLA class II (DR4) transgenic mice (DRAG mice) reconstituted with HLA-matched HSC (38, 39).

In a recent study, we systematically evaluated humanized mice for developing an ultra-sensitive latent viral detection system (Figure 1) (48). First, resting CD4^+^ T cells from HIV-1+ subjects on ART with low, but detectable plasma HIV-1 RNA levels were tested by *in vitro* qVOA to measure the extent of the latent viral reservoir. These samples were positive for viral outgrowth showing a broad range of IUPM levels from 0.102 to 4.468. The CD4^+^ T cells either unstimulated or stimulated *in vitro* with PHA or anti-CD3/CD28 antibodies were injected into humanized mice. Positive viral outgrowth was observed in all of these samples within 1–3 weeks demonstrating the capacity of hu-mice to detect latently infected cells. In some of the patient samples, viral outgrowth was seen with a lesser number of input cells than in the standard qVOA. Stimulation of cells was found to give better viral outgrowth than no stimulation and anti-CD3/CD28 antibody stimulation yielded higher numbers of viable cells for testing compared to that of PHA. To determine if the hmVOA is more sensitive than conventional qVOA, five patient samples that were qVOA negative were tested using a range of CD4^+^ T cells (2–10 million cells/mouse) injected into mice. Of the five qVOA negative patient samples evaluated, four yielded unequivocal positive viral outgrowth in the hmVOA. The earliest time point of viral detection was 2 weeks, whereas the latest time point was 6 weeks. The negative sample did not show any viral outgrowth by 8 weeks, the last time point tested. These observations showed that the hmVOA can detect replication-competent latent HIV-1 when the standard qVOA is unable to do so thus demonstrating the higher sensitivity of this assay. The higher sensitivity of hmVOA over than the *in vitro* qVOA could be attributed to the provision of a more physiological *in vivo* setting for long-term maintenance and expansion of the engrafted cells permitting latency reactivation when compared to the short-term culture of 2 weeks employed *in vitro*.

### Advantages of the hmVOA over the mVOA for Detecting Latent HIV-1

The hmVOA is endowed with higher sensitivity over the mVOA since it was able to detect latent HIV-1 from a higher number of qVOA negative samples and with a fewer number of input cells based on the data published so far (33–36, 48) (Table 1). The higher sensitivity of the hmVOA is likely due to the humanized mice being able to provide more optimal conditions for latent viral outgrowth for several reasons. First, hu-mice generate fresh human HIV-1 targets cells *de novo* (CD4^+^ T cells, monocytes/macrophages, and dendritic cells), including the highly susceptible immature thymocytes thus providing a much broader spectrum of susceptible cells conducive for virus outgrowth. Second, the latently infected cells have the opportunity to home into preformed human lymphoid organs where they can infect cells *in situ* after activation and amplifying the viral signal. Third, hu-mice provide an environment for both xenograft- and allograft-driven cell expansions. Fourth, GvH is almost non-existent in hu-HSC mice and less severe, occurring later in onset, with the BLT mice thus providing a longer time frame (2–3 months) for viral outgrowth. Furthermore, compared to mVOA, no expensive anti-CD8 or anti-CD3 antibody injections are needed after donor cell engraftment.

### Limitations of mVOA and hmVOA and Future Prospects

As discussed above, the *in vivo* mouse-based VOA assays are more sensitive than *in vitro* qVOAs in detecting low levels of HIV-1 latent cells with the hmVOA being the most sensitive. However, these are limitations for these assays to be of wider use. They are not capable of a high-throughput screening, require special animal facilities and are expensive. Nevertheless, due to their higher sensitivity than any *in vitro* tests, they will play an important role in viral latency studies and in “kick/shock and kill” approaches toward a complete cure for HIV/AIDS. These tests will be of utmost benefit *in lieu* of ATI in guiding future curative drug development. Further improvements can be foreseen in the hmVOA and mVOA models with additional research. One approach would be to increase the sensitivity by using HIV-1 LRAs either alone or in various combinations. Thus far, the hmVOA has primarily focused on HIV-1 latency in CD4^+^ T cells. With the recent attention on viral latency in other cell types such as macrophages and work done with SIV latency detection, it is apparent that hmVOA can also be put to good use in evaluating viral outgrowth from HIV-1 latent macrophages as well. Streamlining the hu-mouse generation on a larger scale with increased efficiency should help reduce the overall costs of hmVOA permitting its wider application.

## Author Contributions

Both KS and RA contributed equally in writing and editing this manuscript.

## Conflict of Interest Statement

The authors declare that the research was conducted in the absence of any commercial or financial relationships that could be construed as a potential conflict of interest.
